# Molecular Basis for Genetic Resistance of *Anopheles gambiae* to *Plasmodium*: Structural Analysis of TEP1 Susceptible and Resistant Alleles

**DOI:** 10.1371/journal.ppat.1002958

**Published:** 2012-10-04

**Authors:** Binh V. Le, Marni Williams, Shankar Logarajah, Richard H. G. Baxter

**Affiliations:** Department of Chemistry and Molecular Biophysics & Biochemistry, Yale University, New Haven, Connecticut, United States of America; University of Minnesota, United States of America

## Abstract

Thioester-containing protein 1 (TEP1) is a central component in the innate immune response of *Anopheles gambiae* to *Plasmodium* infection. Two classes of TEP1 alleles, TEP1*S and TEP1*R, are found in both laboratory strains and wild isolates, related by a greater or lesser susceptibility, respectively to both *P. berghei* and *P. falciparum* infection. We report the crystal structure of the full-length TEP1*S1 allele which, while similar to the previously determined structure of full-length TEP1*R1, displays flexibility in the N-terminal fragment comprising domains MG1-MG6. Amino acid differences between TEP1*R1 and TEP1*S1 are localized to the TED-MG8 domain interface that protects the thioester bond from hydrolysis and structural changes are apparent at this interface. As a consequence cleaved TEP1*S1 (TEP1*S1_cut_) is significantly more susceptible to hydrolysis of its intramolecular thioester bond than TEP1*R1_cut_. TEP1*S1_cut_ is stabilized in solution by the heterodimeric LRIM1/APL1C complex, which preserves the thioester bond within TEP1*S1_cut_. These results suggest a mechanism by which selective pressure on the *TEP1* gene results in functional variation that may influence the vector competence of *A. gambiae* towards *Plasmodium* infection.

## Author Summary

*Anopheles* mosquitoes transmit malaria, the world's most devastating parasitic disease, of which *Anopheles gambiae* is the principal vector for malaria in Sub-Saharan Africa. Different populations of mosquitoes vary widely in how readily they become infected with malaria parasites, while some strains do not transmit malaria at all. The mosquitoes' innate immune system is a significant factor that may influence the level of malaria infection; in particular the thioester-containing protein 1 (TEP1) targets malaria parasites for destruction during their initial invasion of the body cavity. The TEP1 gene varies significantly across mosquito populations with two major classes of alleles, TEP1*S and TEP1*R. We report the three-dimensional molecular structure of the TEP1*S1 protein and compare it to the previously determined TEP1*R1 structure. Differences between the structures are localized around the active site and thioester bond, and correlate with a difference in stability of this bond within the two proteins and their interaction with a heterodimer of two other immune genes, LRIM1 and APL1C. These results shed light on the mechanism of mosquitoes' natural immunity to malaria infection.

## Introduction

Thioester-containing proteins (TEPs) are a major component of the innate immune response of insects to invasion by bacteria and protozoa [1], [2]. *Anopheles gambiae* thioester-containing protein 1 (TEP1) is a complement-like protein that plays a central role in the opsonization of gram-negative bacteria in the hemolymph [3]. TEP1 also binds to the surface of *Plasmodium* ookinetes that traverse the midgut epithelium following ingestion of an infectious blood meal, targeting those ookinetes for lysis and, in certain mosquito strains, melanization [4]. TEP1 activity has been demonstrated against both *P. berghei* [4] and *P. falciparum* [5], [6]. Deciphering the molecular basis of the TEP1-mediated immune response is relevant to understanding the determinants of vector competence and a potential source of novel vector-based malaria control strategies.

The crystal structure of full-length TEP1 revealed significant structural homology to complement factor C3 [7]. TEP1 is composed of a series of eight macroglobulin (MG) domains, the β-sheet CUB domain and α-helical thioester domain (TED). The TED contains an intramolecular β-cysteinyl-γ-glutamyl thioester bond that is protected from inadvertent hydrolysis by sequestration within a protein-protein interface formed by the TED and MG8 domains. Based upon the known mechanism of complement factors [8], activation of the thioester in TEP1 is presumed to involve a large conformational change causing dissociation of the TED-MG8 interface in the direct proximity of a pathogen, whereupon the thioester may react with nucleophilic groups on, and covalently attach TEP1 to, the surface of the pathogen.

TEP1 lacks two additional domains that are present in complement factors, the anaphylatoxin (ANA) and C345C domains. The ANA domain in particular plays a key role in the activation of complement factor C3, which is cleaved intracellularly in a protease-sensitive region between the MG6 and ANA domains prior to secretion. The ANA domain contacts both the MG3 and MG8 domains in the structure of mature, circulating C3 [9]. Activation of C3 occurs after regulated proteolysis immediately following the ANA domain whereby the anaphylatoxin C3a is released. Dissociation of C3a destabilizes the remaining C3b fragment and leads to a large-scale conformational change and rapid activation of the thioester bond [10], [11].

In contrast, TEP1 is secreted as a full-length protein into the mosquito hemolymph where it is cleaved by as yet unknown protease(s). Cleavage of TEP1, producing TEP1_cut_, does not instantly lead to activation of the thioester [12], suggesting that full-length TEP1 is a pro-form [13] that must undergo conversion to an active species following cleavage within the protease-sensitive region. TEP1_cut_ is meta-stable in solution and precipitates over time. This precipitation is concomitant with hydrolysis of the thioester bond and is prevented *in vivo* by formation of a ternary complex between TEP1_cut_ and a heterodimer of two leucine-rich repeat proteins, LRIM1 and APL1C [12], [14]–[16]. The ternary complex TEP1_cut_/LRIM1/APL1C was formed *in vitro* only after chemical inactivation of the thioester bond of TEP1_cut_ by treatment with methylamine (MeNH_2_) [15]. This raised the question as to whether LRIM1/APL1C stabilizes a conformation of TEP1_cut_ that contains an active thioester, or a distinct conformation in which the thioester has either reacted with substrate or been hydrolyzed by water.

The *TEP1* gene is highly polymorphic, with distinct alleles conferring variable levels of protection from pathogens. Two alleles were originally identified in laboratory mosquito strains (indicated in brackets) as being susceptible (G3) and refractory (L3–5) to infection with *P. berghei* [4]. Recently, additional alleles were identified from laboratory strains conforming to two major classes S and R: TEP1*S1 (PEST), TEP1*S2 (4Arr), TEP1*S3 (G3), TEP1*R1 (L3–5) and TEP1*R2 (4Arr) with TEP1*S2 and TEP1*R2 alleles displaying intermediate phenotypes with respect to *P. berghei* infection [17]. The refractory allele, TEP1*R1, has been expressed *in vitro* and utilized in structural and functional studies [7], [12], [15]. The TEP1*S1 and TEP1*R1 proteins share 93% sequence identity with the majority of amino acid differences being confined to three hypervariable loops within the TED domain [3], [4]. Two of these loops, the pre-α4 loop and the catalytic loop, are situated in close proximity to the thioester itself at the TED-MG8 domain interface [7] and are complemented by amino acid differences within the MG8 domain that interact with the pre-α4 and catalytic loops and also conform to the TEP1*S/R division of alleles.

A recent study of wild mosquito populations from five locations in West, Central, and East Africa detected three similar sets of TEP1*S/R alleles as observed in the laboratory strains; *s* (TEP1*S), *r^A^* (TEP1*R2) and *r^B^* (TEP1*R1) [6]. Furthermore, specific geographical variation in allelic frequencies and a statistically significant decrease in *P. falciparum* oocysts within *s*/*r^B^* heterozygous vs. *s*/*s* homozygous mosquitoes were observed. The concordance of laboratory and field studies prompted us to further investigate the structure and properties of TEP1*S1 in comparison to TEP1*R1. Here we report the crystal structure of full-length TEP1*S1. We also report the relative reactivity of the thioester bond in TEP1*S1 and TEP1*R1 to hydrolysis and the association of TEP1*S1_cut_ with LRIM1/APL1C. These results suggest a potential mechanism by which allelic variation in *TEP1*, particularly in the pre-α4 and catalytic loops, may translate to functional variation towards distinct pathogens.

## Results

### Quaternary structure of full-length TEP1*S1

Full-length TEP1*S1 crystallized in space group *P*4_3_ and the structure was determined at 3.7 Å resolution (PDB 4D93), (see Materials and Methods, Table S1 and Figure S1). To facilitate comparison with recent studies of *TEP1* alleles the structure of TEP1*S1 is numbered according to the complete protein sequence. The previously determined structure of TEP1*R1 [7] has been re-refined (PDB 4D94) to correct some errors in the original model and was used as a reference structure for refinement of TEP1*S1. Residues in the new TEP1*R1 and TEP1*S1 models are numbered according to the complete peptide chain (including signal peptide) for comparison with other reports.

The refined model of TEP1*S1 has three molecules, two of which (chains A, C) comprise residues 22–1338 with the exception of four gaps; residues 561–562, 575–582 in the linker (LNK) domain, 606–628 within the protease-sensitive region, and 822–829 in the CUB domain. A third molecule (chain B) with higher *B*-factors has poor or absent density for much of domains MG1, MG2, MG4, MG5 and MG6, but is otherwise complete for residues 629–1338 with the exception of 822–829 in the CUB domain. No significant difference in conformation is apparent between the three molecules. Further description of the structure is based upon molecule A.

**Figure 1:**
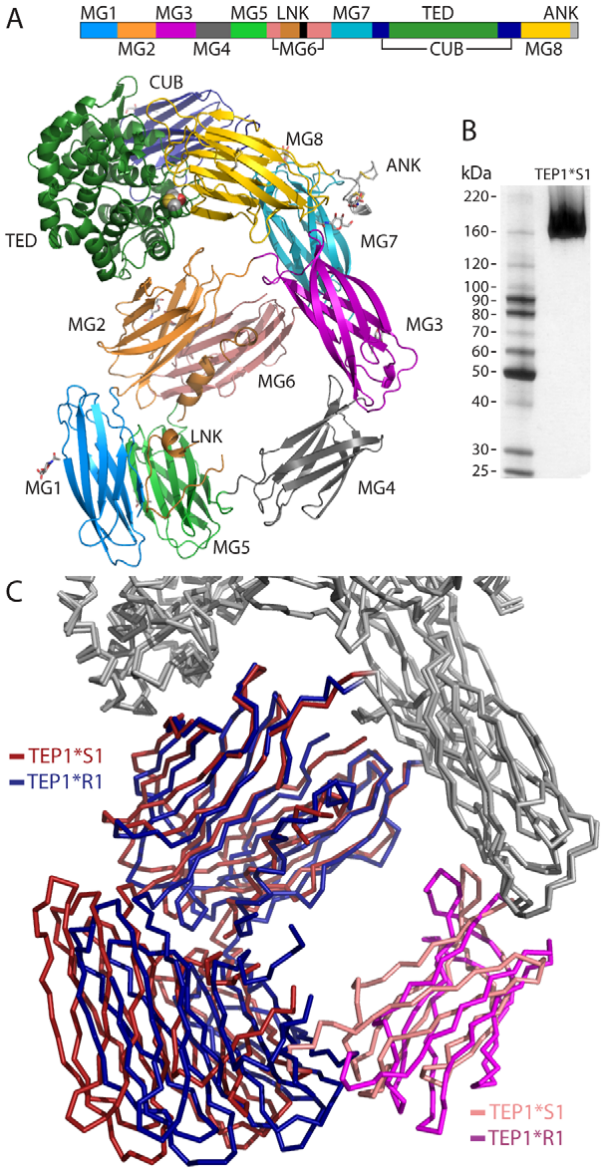
Overview of TEP1*S1 structure. (A) Sequence schematic and three-dimensional structure of TEP1*S1. The mature protein commences with domain MG1 (mid-blue) followed by MG2 (orange), MG3 (purple), MG4 (mid-grey), MG5 (light green), MG6 (pink), LNK (light brown), MG7 (light blue), CUB (navy blue), TED (dark green), MG8 (yellow) and ANK (light grey). (B) Silver-stained SDS-PAGE of redissolved crystals confirms structure corresponds to full-length TEP1*S1. (C) Rigid body domain motions between TEP1*S1 and TEP1*R1. The MG1–2 and MG5–6 domains (*S1 red, *R1 blue) rotate by 11° relative to the remainder of the protein, with the MG4 domain (*S1 pink, *R1 magenta) acting as a hinge.

The overall structure of TEP1*S1 is very similar to that of TEP1*R1 (Figure 1A). The first six MG domains form a super-helical quaternary structure, with MG6 split by the insertion of the linker and protease-sensitive regions (585–607). Thus the TEP1_cut_ N-terminal fragment (β chain) and C-terminal fragment (α chain) are interleaved within the MG6 domain. Following the MG6 domain two additional domains, MG7 and MG8, are divided by the nested insertions of the CUB and TED domains. SDS-PAGE of redissolved crystals confirms TEP1*S1 within the crystal to be full-length protein (Figure 1B). Some protein domain motion is evident between TEP1*S1 and TEP1*R1 (Figure 1C), confirmed by analysis using the program *DYNDOM* [18] (Table S2). The TEP1*S1 MG3, MG7, CUB, TED, and MG8 domains are superimposable as a rigid body upon TEP1*R1. The MG1, MG2, MG5 and MG6 domains also form a rigid body but are rotated 11° relative to TEP1*R1. One hinge for this movement is the MG2–3 linker (217–222) a short sequence identically conserved with human complement factor C3. The second hinge is the MG4 domain itself which is rotated 26° relative to the two other rigid domains. As there is no sequence variation between TEP1*S1 and TEP1*R1 at the interface of the MG2, MG6 and TED domains or the MG3–MG4 domain interface, these rearrangements likely reflect inherent flexibility and different packing constraints within the TEP1*S1 crystal vs. TEP1*R1.

Amino acid variation between TEP1*R1 and TEP1*S1 was previously noted to be largely confined to domains surrounding the TED [4], [7]. Analysis of alleles TEP1*R1–2 and TEP1*S1–3 [17] confirms and extends this observation. No amino acid substitutions that separate TEP1*R and TEP1*S alleles occur within domains MG1–MG6, and except for five substitutions to similar residues, all variation between TEP1*S and TEP1*R alleles are confined to the TED, CUB and MG8 domains. These polymorphisms are hereafter described as mutations to the TEP1*R1 allele, i.e. *R*{res#}*S*.

### Variation within the TED

We focused on amino acid differences between TEP1*S1 and TEP1*R1 that are preserved in all *S* and *R* laboratory alleles [17] and wild mosquito populations [6]. Of 42 such polymorphisms within the TED (Table S3), 18 occur within three previously identified hypervariable loops termed the pre-α4 loop (914–920), the catalytic loop (966–974) and the β-hairpin (1054–1069). An additional polymorphism S1108R was noted from the crystal structure of TEP1*R1 as potentially significant [7]. The remaining 23 polymorphisms are generally localized in short loops between the TED α-helices and introduce no significant alteration to the structure (Table S3), with the possible exception of four (F960S, E1005V, K1009V, T1012N) on the face of helix α7 and adjacent to the post-α5 turn that form a crystal contact within the TEP1*R1 structure.

**Figure 2:**
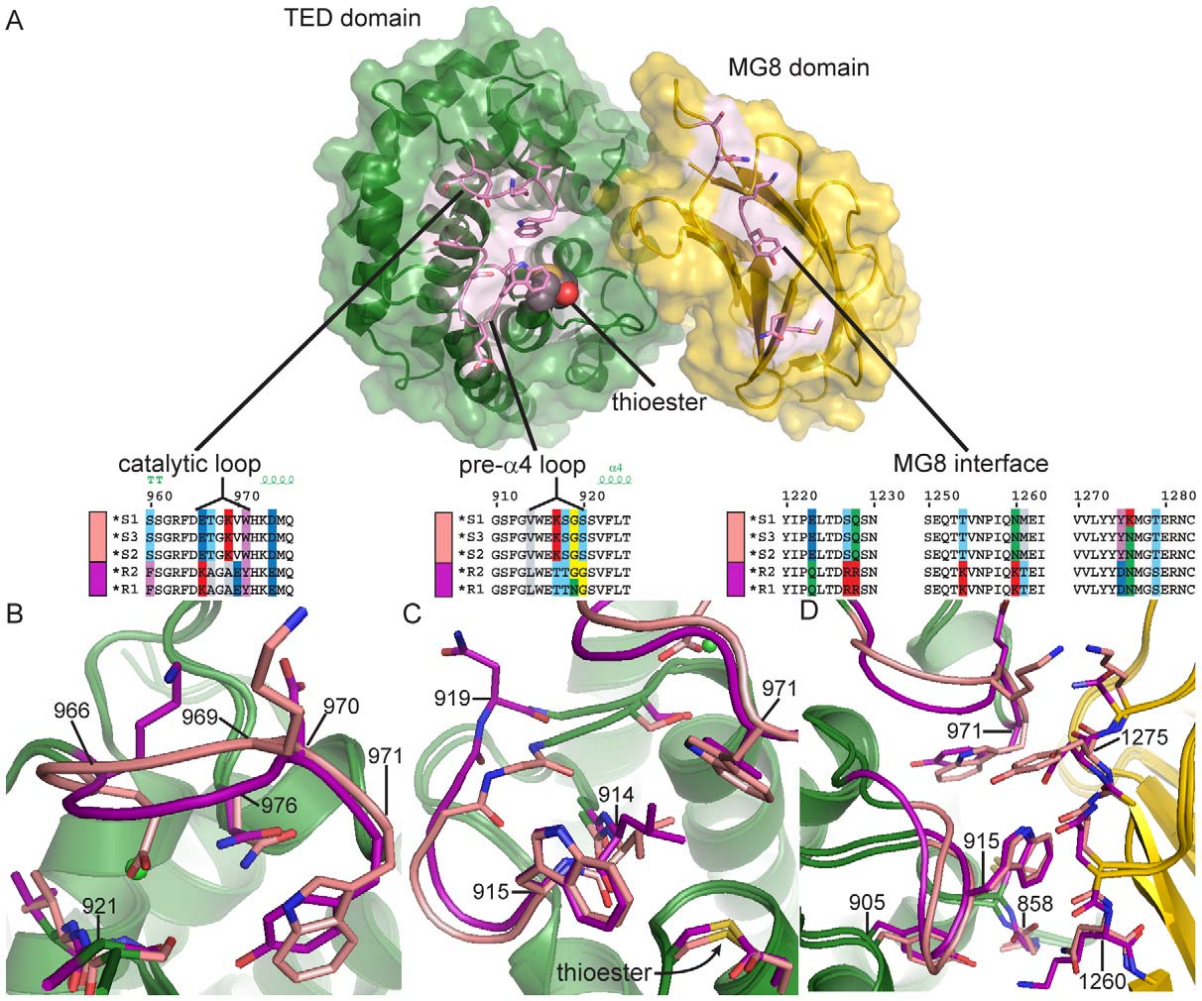
Comparison of TEP1*S1 and TEP1*R1 TED-MG8 interface. (A) Exploded view of the TED-MG8 interface, the MG8 domain (yellow) has been rotated 90° with respect to the TED (green). The thioester bond is shown as VDW spheres, variable residues within the pre-α4 loop, catalytic loop and MG8 interface (pink) are shown as sticks. Sequence alignments for TEP1 alleles in each variable region are illustrated by superposition of TEP1*S1 (pink) and TEP1*R1 (magenta) structures in panels (B–D) with non-variable regions colored by domain for both alleles. (B) The catalytic loop (966–976); TEP1*S1 Glu 966 is directed towards Ser 921 in place of TEP1*R1 Tyr 971, causing a 2 Å displacement of residues 967–979. (C) The pre-α4 loop (914–920); TEP1*S1 Gly 919 is directed towards Val 914 whereas TEP1*R1 Asn 919 is directed towards the solvent, (3.9 Å displacement of C_α_). (D) Complementary variation within the MG8 domain; TEP1*S1 Asn 1260 is within hydrogen bonding distance of Gly 858 but TEP1*R1 Lys 1260 also interacts with Tyr 884, and TEP1*S1 Tyr 1275 is not compatible with hydrogen bonding to Trp 915 as is the case for TEP1*R1 Asn 1275.

The pre-α4 and catalytic loops form part of the TED-MG8 domain interface (Figure 2A) that protects the thioester from premature activation or hydrolysis. Both loops are ordered in the TEP1*S1 structure. The TEP1*R1 catalytic loop contains a sequence of five residues including Lys 966 and Glu 970 (^966^KAGAE^970^). These charged residues also occur in the TEP1*S1 catalytic loop but their positions are switched (^966^ETGKV^970^). TEP1*S1 Glu 966 adopts a different conformation than Lys 966 in TEP1*R1 (Figure 2B), directed into a pocket occupied by a Cl^−^ ion in TEP1*R1, within hydrogen bonding distance of Ser 921 O_γ_ and Phe 923 N (the same conformation observed for Glu 1098 in complement factor C3). In contrast Ser 921 is within hydrogen bonding distance of TEP1*R1 Tyr 971 but not TEP1*S1 Trp 971. These differences impart a ∼1.8 Å displacement of residues 966–968 in TEP1*S1 relative to TEP1*R1.

In the pre-α4 loop the substitution N919G permits a different backbone conformation for TEP1*S1 with Gly 919 O within hydrogen bonding distance of Val 914 N (Figure 2C). The L914V and S1108R substitutions were previously noted as potentially affecting the environment of the thioester [7]. The displacement of the catalytic loop in TEP1*S1 leads L914V to introduce a small cavity within the TED-MG8 interface between Trp 971 and the thioester bond. The S1108R substitution does not cause any perturbation in the interface however, the Arg side chain adopts a conformation within hydrogen bonding distance of the carbonyl oxygen of Tyr 1307 instead of a water molecule as seen in TEP1*R1.

### Variation within the MG8 domain

Substitutions in the TED domain at the TED-MG8 interface are complemented by substitutions within the MG8 domain (Table S4). Three pairs of substitutions noted in the TEP1*R1 structure [7] are preserved between the TEP1*S and TEP1*R alleles, two of which produce significant differences in the TEP1*S1 structure (Figure 2D). The K1260N substitution preserves the hydrogen bonding distance to Gly 858 N in the thioester motif but not to Tyr 884. The N1275Y substitution is no longer compatible with hydrogen bonding to Trp 915 in the pre-α4 loop, and the conformation adopted by TEP1*S1 Tyr 1275 forms is neither favorable for alternative hydrogen bond formation nor π-stacking interactions with nearby aromatic residues. Though the substitution N1276K appears to introduce a repulsive electrostatic interaction with Lys 970 in the TEP1*S1 catalytic loop we note that (i) the density for this side chain is poor, (ii) the nearby substitutions R1227S/R1228Q compensate for the introduction of this charge and (iii) Asn is conserved at this position in TEP1*S2–S3 [17] (the corresponding region was not sequenced for wild alleles reported by White et al. [6]).

### Other variation between TEP1*R1 and TEP1*S1

An additional 11 polymorphisms conserved between *S* and *R* alleles occur in the MG8 domain but introduce no discernible alterations (Table S4). Amino acid variation within the CUB domain is localized to peripheral residues, none of the *S*/*R*-conserved polymorphisms are observed in the central β-strands β5, β6, β7 or β10 (Table S5). Two pairs of substitutions, V797A/R800K and V1183I/N1187D, are located on adjacent strands linking the CUB domain to the MG7 and MG8 domains, respectively, with the substitution T831K adjacent at the end of the β4–β5 turn. This is a site of large structural changes in the conversion of complement factor C3 to C3b [10], [11], and the site of C3b cleavage by factor I [19].

### Reduced stability of TEP1*S1_cut_ in solution

**Figure 3:**
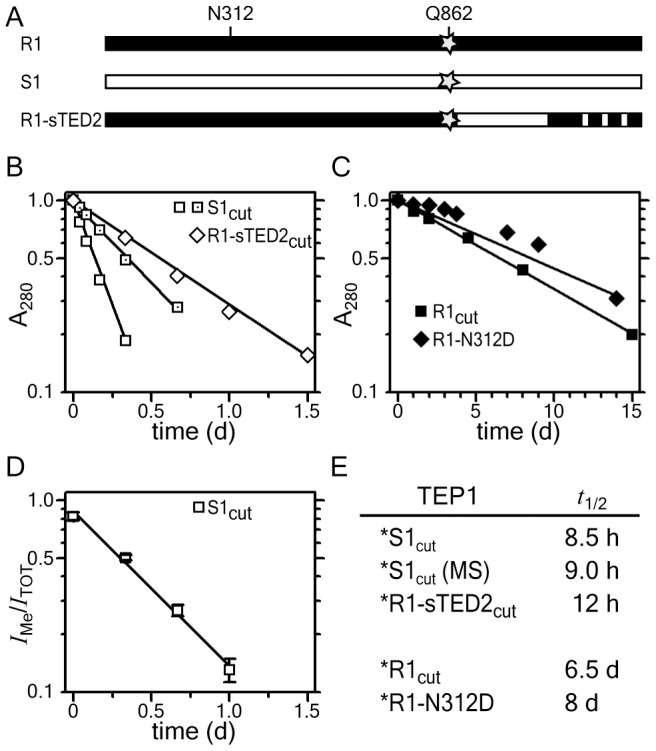
Rate of TEP1_cut_ thioester hydrolysis. (A) Schematic diagram of TEP1*R1, TEP1*S1 and the chimera TEP1*R1-sTED2, and location of thioester Gln 862 (star) and MG3 Asn 312. (B–C) Rate of TEP1_cut_ precipitation. (B) TEP1*S1_cut_ (open squares) and TEP1*R1_cut_-sTED2 (open diamonds) precipitate within 1.5 days, while (C) TEP1*R1_cut_ (squares) and TEP1*R1_cut_-N312D (diamonds) precipitate over several weeks. (D) Rate of thioester hydrolysis for TEP1*S1 (open squares) by quantitative mass spectrometry. (E) Measured half-life for thioester hydrolysis of TEP1 proteins.

To assess the functional role of TEP1 polymorphisms *in vitro*, we sought to determine the relative stability of the TEP1*S1 compared to TEP1*R1. In addition to wild-type alleles we generated the following TEP1 variants: (i) TEP1*R1 with thioester cysteine mutation C859A, (ii) TEP1*R1-sTED2, in which residues 878–1108 in the TED and 1227–28, 1260–61 and 1275–76 in MG8 were replaced with TEP1*S1, and (iii) TEP1*R1 with MG3 glycosylation mutant N312D (Figure 3A). We previously observed that limited proteolysis of TEP1 in the protease-sensitive region leads to slow hydrolysis of the thioester bond and, in the absence of the LRIM1/APL1C complex, hydrolysis of the thioester leads to precipitation [15]. We purified TEP1*R1-C859A and observed that, while it was a stable full-length protein, the protein precipitated rapidly following proteolysis (Figure S2), suggesting that thioester hydrolysis is the rate-limiting step in the precipitation of TEP1_cut_.

We therefore measured the rate of precipitation of TEP1*R1_cut_ and TEP1*S1_cut_ to determine the rate of thioester hydrolysis in TEP1_cut_ at 20°C, the same temperature used for *in vivo* studies of *P. berghei* infection. The half-life of TEP1*S1_cut_ is 8.5 h (Figure 3B), significantly shorter than the half-life of TEP1*R1_cut_ (6.5 days), suggesting that TEP1*S1_cut_ is more susceptible to hydrolysis of the thioester bond than TEP1*R1_cut_ (Figure 3C). The soluble fractions of the TEP1_cut_ proteins analyzed with silver-stained SDS-PAGE also reflects the shorter half-life of TEP1*S1_cut_ (Figure S3). The half-life of TEP1*R1-sTED2_cut_ is 12 h (Figure 3B), confirming that the increased reactivity of TEP1*S1 towards hydrolysis is largely due to variation within the TED domain and the TED-MG8 interface in particular. The glycosylation site Asn 312 was previously noted to form a significant fraction of the interface between the MG3 and MG8 domains [7]. The half-life of the glycosylation mutant TEP1*R1_cut_-N312D is 8 days however (Figure 3C), indicating that removal of this glycosylation site does not affect the stability of the thioester in TEP1*R1_cut_.

Precipitation of TEP1_cut_ is an indirect effect of thioester hydrolysis and may not correlate quantitatively with the rate of reaction of the thioester. We therefore measured the fraction of TEP1*S1_cut_ containing an intact thioester by treatment of samples with MeNH_2_ as a function of time. Methylated and hydrolyzed TEP1_cut_ were simultaneously quantified by monitoring the modification of Gln 862 with quantitative mass spectrometry (see Materials and Methods). The fraction of methylated TEP1*S1 decreased with time with an estimated half-life of 9 h (Figure 3D), in close agreement with the rate of precipitation of the protein. This supports the conclusion that hydrolysis of the thioester bond is the rate-limiting step in precipitation of TEP1_cut_.

### Ternary complex of TEP1*S1_cut_ with LRIM1/APL1C

**Figure 4:**
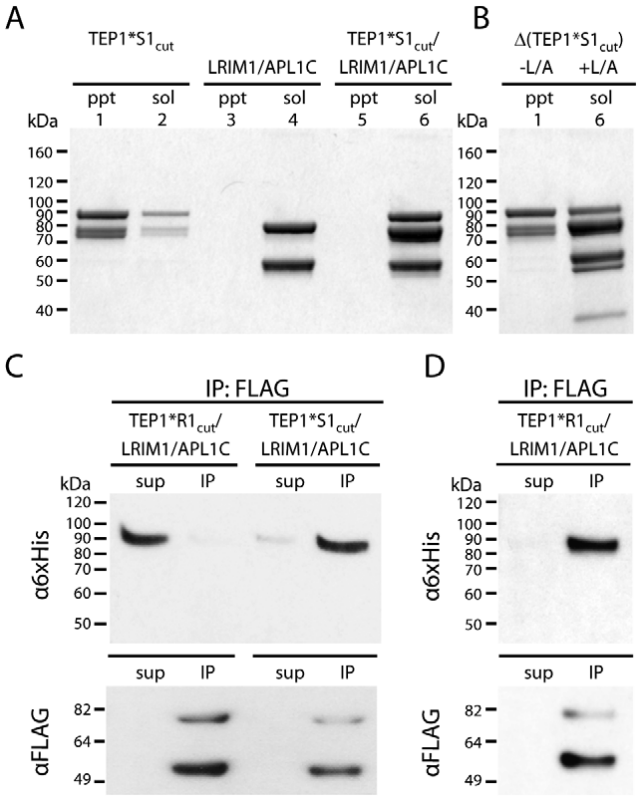
Formation of ternary complex between TEP1*S1_cut_ and LRIM1/APL1C. (A) Insoluble (ppt) and soluble (sol) fractions of TEP1*S1_cut_ (lanes 1–2), LRIM1/APL1C (3–4) and TEP1*S1_cut_/LRIM1/APL1C (5–6) after 36 h at 20°C. (B) Autolytic cleavage assay (Δ) shows the thioester bond in insoluble TEP1*S1_cut_ (1) is hydrolyzed while soluble TEP1*S1_cut_ in complex with LRIM1/APL1C (6) has an intact thioester (heat-induced cleavage of TEP1 C-terminal 85 kDa band). (C) FLAG co-immunoprecipitation of TEP1*R1_cut_ and TEP1*S1_cut_ with LRIM1/APL1C after 48 h (TEP1*R1_cut_) and 24 h (TEP1*S1_cut_), and (D) after 24 days (TEP1*R1_cut_). Proteins in both the supernatant (sup) and immunoprecipitated (IP) samples were detected with Western blotting using either α6×His antibody to detect APL1C-6×His and TEP1-6×His C-terminal chain, or αFLAG antibody to detect LRIM1-FLAG/APL1C-FLAG.

We previously observed the ternary complex TEP1_cut_/LRIM1/APL1C was formed only after chemical inactivation of the thioester bond of TEP1*R1_cut_ by MeNH_2_ [15], demonstrating that LRIM1/APL1C interacted with a reacted form of TEP1*R1_cut_ without an intact thioester. To test whether this was also the case for TEP1*S1, we prepared TEP1*S1_cut_ and incubated for 36 h at 20°C in the absence or presence of LRIM1/APL1C (Figure 4A). TEP1*S1_cut_ incubated without LRIM1/APL1C precipitated (Figure 4A, lanes 1–2), while TEP1*S1_cut_ mixed with LRIM1/APL1C remains soluble (Figure 4A, lanes 5–6).

The presence of an intact thioester bond in thioester-containing proteins can be determined by heating under denaturing conditions in the absence of reducing agent, promoting autolytic cleavage of the peptide chain at the site of the thioester bond [20]. The thioester bond is hydrolyzed in precipitated TEP1*S1_cut_ (Figure 4B, lane 1). In contrast, soluble TEP1*S1_cut_ in complex with LRIM1/APL1C possesses an intact thioester, as shown by heat-induced fragmentation of the C-terminal fragment (Figure 4B, lane 6). We conclude that the conformational changes in TEP1 required for the binding of LRIM1/APL1C is distinct from that involving reaction of the thioester. Hence the complex between LRIM1/APL1C and TEP1*S1_cut_ is a distinct species from the complex of LRIM1/APL1C and TEP1*R1_cut_(MeNH_2_) [15].

The preceding experiments suggest that formation of the ternary complex between TEP1*S1_cut_ and LRIM1/APL1C is due to a conformational change with a similar half-life as the measured rate of thioester hydrolysis. Thus previous attempts to produce a ternary complex between TEP1*R1_cut_ and LRIM1/APL1C were unsuccessful simply because the period of incubation was too short. Accordingly FLAG immunoprecipitation assays were performed with 6×His-tagged TEP1_cut_ proteins and FLAG-tagged LRIM1/APL1C. TEP1*S1_cut_ co-immunoprecipitated with LRIM1/APL1C within 24 h (∼3× *t*_1/2_ for thioester hydrolysis) whereas TEP1*R1_cut_ remained in the supernatant after 48 h (Figure 4C). However, after incubation at 20°C for 24 days (∼4× *t*_1/2_ for thioester hydrolysis) TEP1*R1_cut_ remained soluble and was co-immunoprecipitated with LRIM1/APL1C (Figure 4D). Thus the conformational change following limited proteolysis *in vitro* that allows TEP1*S1_cut_ and TEP1*R1_cut_ to bind LRIM1/APL1C is comparable to their respective rates of thioester hydrolysis and precipitation in the absence of LRIM1/APL1C.

## Discussion

As a central component of humoral immunity in *A. gambiae*, the *TEP1* gene is under selective pressure. Significant variation within two major allelic forms, TEP1*S and TEP1*R, are found in both laboratory and wild mosquito populations. Comparison of the structures of TEP1*S1 and TEP1*R1 reveals the consequences of this variation on the pro-form of TEP1 and stabilization of the intramolecular thioester bond. We observe distinct side chain and backbone conformations of two hypervariable loops within the thioester domain and two complementary substitutions within the MG8 domain that directly influence the TED-MG8 interface and the surrounding environment of the thioester bond.

An important caveat in analysis of the present structures is that the role of specific polymorphisms may be relevant to another conformation of TEP1 than is represented in the full-length protein. At present three soluble forms of TEP1_cut_ have been identified *in vitro* ([12], [15] and this study). The first form contains an intact thioester and does not bind LRIM1/APL1C, (e.g. TEP1*R1_cut_ 0–48 h post-cleavage). The second form contains a thioester but requires LRIM1/APL1C for stability in solution (e.g. TEP1*S1_cut_ 24–36 h post-cleavage). The third form does not contain a thioester and also requires binding of LRIM1/APL1C for stability in solution (e.g. TEP1*R1_cut_(MeNH_2_) 12 h post-cleavage).

Distinct phenotypes for TEP1*S and TEP1*R alleles are observed for the response to both *P. berghei* [4] and to *P. falciparum* [6]. Our results provide the first evidence for a distinct chemical property of TEP1*S and TEP1*R proteins; the rate of thioester hydrolysis and precipitation in the absence of LRIM1/APL1C. Furthermore this difference affects the relative amount of the three *in vitro* soluble TEP1_cut_ forms arising from cleavage in the protease-sensitive region. Within 24–36 h post-cleavage at 20°C the major soluble form of TEP1*S1_cut_ has an intact thioester and binds LRIM1/APL1C, whereas TEP1*R1_cut_ has an intact thioester but does not bind LRIM1/APL1C. In the absence of LRIM1/APL1C ∼90% of TEP1*S1_cut_ has undergone hydrolysis of the thioester and precipitated from solution within 24 h at 20°C, whereas ∼90% TEP1*R1_cut_ has an intact thioester bond and is soluble [12]. Hence, our results suggest that phenotypic variation in *TEP1* alleles can result not only by activity in a single pathway but by distinct mechanisms arising from different forms present in the hemolymph.

**Figure 5:**
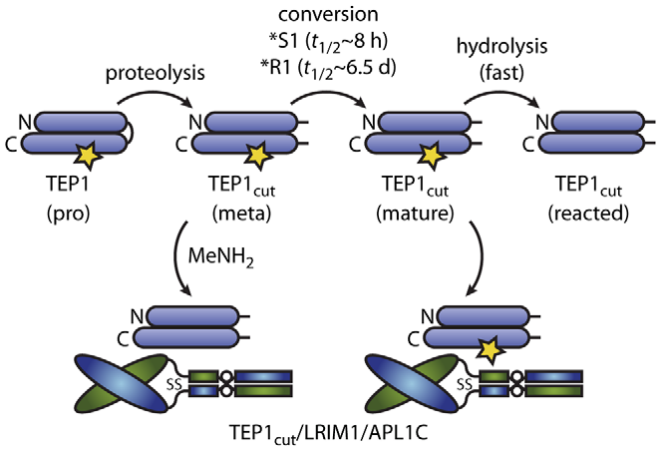
Conformational change and thioester reactions of TEP1*S1 and TEP1*R1 *in vitro*. TEP1 N- and C-terminal fragments are shown as horizontal ovals linked by the protease-sensitive region, the thioester bond is indicated by a star. Full-length TEP1 is secreted in a pro-form. Limited proteolysis in the hemolymph to a meta form is followed by a slow conversion to an active form. The LRIM1/APL1C pathway involves capture and stabilization of the mature form of TEP1*S1. Methylamine (MeNH_2_) was previously used to produce a ternary complex of reacted TEP1*R1_cut_ with LRIM1/APL1C [15].

Our *in vitro* studies may directly pertain to *in vivo* studies of *P. berghei* infection that are also conducted at ∼20°C [21] with microscopic analysis of TEP1 binding at 24–48 hours post-infection [4], [12], [22]. Our results are consistent with a model for activation of TEP1*S as proposed by Fraiture et al. (2009) [12] (Figure 5). Full-length TEP1*S represents a pro-form. Cleavage within the protease-sensitive region produces a meta-stable species similar to the pro-form that does not interact with LRIM1/APL1C. A slow (8 h) spontaneous conformational change generates a mature form of TEP1*S and exposes a cryptic binding site for LRIM1/APL1C. In the absence of LRIM1/APL1C however, the thioester bond in the mature form is susceptible to hydrolysis, presumably coupled to a large conformational change, producing a reacted form that rapidly aggregates and precipitates from solution. This model is consistent with the roles of TEP1*S3, LRIM1 and APL1C in the immune response of *A. gambiae* G3 to *P. berghei* ookinetes [4], [12], including the concept of basal immunity [22], as spontaneous formation of the active immune complex TEP1*S3_cut_/LRIM1/APL1C at 20°C is slow relative to the residence of ookinetes beneath the basal lamina.

TEP1*R1_cut_ also forms a complex with LRIM1/APL1C that presumably contains an active thioester by a spontaneous conformational change with a half-life of 6.5 days at 20°C (Figure 5). We previously observed that TEP1*R1_cut_ retained an active thioester after 60 h incubation with LRIM1/APL1C and a small component of a high molecular weight complex with LRIM1/APL1C [15]. Originally interpreted as hydrolysis of TEP1*R1_cut_, this may now be considered to be slow formation of the same complex formed by TEP1*S1_cut_. Such a slow rate of complex formation however, cannot account for the LRIM1/APL1C-dependent activity of TEP*R1 against *P. berghei* ookinetes [14] that traverse the midgut epithelium 18–24 h post blood-meal. Hence additional factor(s) must exist that accelerate the conformational change of TEP1*R1_cut_ *in vivo*.

In the context of the present model, such factor(s) could act in three ways. First, protease(s) that cleave TEP1 may comprise or recruit chaperone(s) that accelerate the maturation of TEP1, revealing the binding site for LRIM1/APL1C; TEP1 activation would remain LRIM1/APL1C-dependent. Second, factors could accelerate both maturation and activation of TEP1 directly in an LRIM1/APL1C-independent manner. Third, factors may interact with a complex of reacted TEP1_cut_ and LRIM1/APL1C to activate other TEP1 molecules, i.e. a “TEP1 convertase” as proposed previously to explain the interaction of TEP1*R1_cut_(MeNH_2_) with LRIM1/APL1C [15] (Figure 5).

Distinct TEP1*S/*R* phenotypes are observed for both the LRIM1/APL1C-dependent response to *P. berghei* [12], [14] and the response to *P. falciparum* that is LRIM1/APL1C-independent in Yaoundé and Ngousso strains [23], [24] that carry TEP1*S alleles [6]. This suggests a functional role of TEP1*S/R polymorphisms in the active form of TEP1, i.e. direct interaction of the pre-α4 and catalytic loops with the thioester and pathogen surfaces at the point of covalent attachment. The selective pressure that has given rise to these polymorphisms is not only (even unlikely) *Plasmodium*, but environmental pathogens such as bacteria encountered in both adult and pre-adult stages. Our results suggest a possible trade-off between selection for reactivity of the thioester upon activation and steady-state stability of the thioester in circulating TEP1_cut_. This may be relevant to immune responses based upon basal immunity, as is indicated in the case of *Plasmodium* [22], compared to responses based upon infection-induced upregulation of TEP1 expression.

Many outstanding questions remain regarding the mechanism of TEP1-mediated immune responses. The structure of the TEP1_cut_/LRIM1/APL1C ternary complex and the interaction of LRIM1/APL1C with reacted TEP1*R1_cut_ [15], the source of phenotypic differences between different TEP1*S and TEP1*R alleles, and the role of polymorphism in the TED β-hairpin remains unknown. The interaction of LRIM1/APL1C with MeNH_2_-treated TEP1*R1_cut_ [15] and with distinct TEP proteins TEP3 and TEP4 [16] suggest additional roles for LRIM1/APL1C in TEP1-mediated immunity besides stabilization of a re-circulating active immune complex. Further structural and functional studies of TEP1, LRIM1/APL1C and the identification of additional factors are required to address these questions.

## Materials and Methods

### Protein expression and purification

TEP1*S1 was generated by total gene synthesis (Genscript) and subcloned into pFastbac1 with a C-terminal 6×His tag. TEP1*R1-sTED2 was constructed as follows using QuickChange site-directed mutagenesis (Stratagene). An *Sph*I site was inserted into TEP1*R1-pFastbac1 corresponding to TEP1*S1 H878Y and removed from the pFastbac1 MCS. Digestion of both vectors with *Sph*I/*Afe*I allowed replacement of TEP1*R1 residues 878–1108 with the corresponding sequence from TEP1*S1. Finally (i) 1227–8, 1260–1 and 1275–6 in the MG8 domain (TED-MG8 interface) were mutated to the corresponding residues in TEP1*S1, and (ii) residues 960, 1005, 1009, 1012 in the TED were mutated back to the corresponding residues of TEP1*R1. All TEP1 and LRIM1/APL1C constructs were expressed using the baculovirus expression system. Purification, limited proteolysis, thioester autolytic cleavage assay and immunoprecipitation experiments were performed as previously described [7], [12], [15].

### Thioester hydrolysis precipitation assay

Following limited proteolysis and re-purification TEP1 samples were concentrated to an OD_280_ of 0.5–1.0 and stored at 20°C. To measure rate of precipitation as a result of thioester hydrolysis upon proteolysis, samples and matching blank (filtrate from concentration) were centrifuged at 17,000×*g*, 20°C for 10 min and *A*_280_-*A*_330_ recorded in a standard UV spectrophotometer (Shimadzu UV1800). Separate time points are all derived from the same protein batch and purification and qualitatively similar results derived from 2–3 independent biological replicates. Half-lives were calculated from samples with a decay to <25% initial value and fit to log-linear plot with *R* ^2^>0.99 (except TEP1*R1-N312D, final value 30% initial, *R* ^2^ = 0.95).

### Thioester hydrolysis LC-MS assay

To determine the rate of thioester hydrolysis by quantitative mass spectrometry, TEP1*S1 was cleaved as before and MeNH_2_ was added at specific time points to react with intact thioester bonds, methylating Gln 862. Samples were TCA precipitated and redissolved in 0.4 M NH_4_HCO_3_ containing 8 M urea followed by reduction and alkylation with DTT and iodoacetamide, respectively. Trypsin digestion was performed for 16 h at 37°C at a 10-fold molar excess of protein to trypsin. TFA and acetonitrile was added to final concentrations of 0.5% and 5%, respectively, followed by purification with C18 spin columns (Pierce) and elution in 80% acetonitrile. Tryptic peptides corresponding to hydrolysis (deaminated, [Dea] = +1 *m/z*) or methylation ([Me] = +14 *m/z*) of TEP1*S1 Gln 862 were characterized by time-of-flight LC-MS. Three specific fragments selected for quantitative analysis on an AB SCIEX 5500 Q-TRAP instrument coupled to an online Waters nanoACQUITY Ultra High Pressure Liquid Chromatography system and analysis with Multiquant 2.0 software. Assuming the deaminated and methylated peptides have similar ionization efficiency, the fraction of intact thioester is equal to *I*_Me_/(*I*_Me_+*I*_Dea_). Reported data is the average of three fragments, two instrument replicates.

#### TEP1*S1 crystallization

Initial crystals of TEP1*S1 were obtained for 4 mg/ml protein in 30% PEG400, 0.1 M Li_2_SO_4_, 0.1 M Na citrate pH 5.6. Diffraction quality crystals grew over several weeks in 27% PEG400, 0.1 M Li_2_SO_4_, 0.1 M Na citrate, 1% 1,2-butanediol. Crystals were extremely fragile under conditions necessary for cryopreservation, optimal diffraction was obtained by overnight equilibration with 29% PEG400 and 20% glycerol prior to freezing directly in a nitrogen cryostream at 100 K.

#### Data collection and refinement

X-ray data was collected at NSLS X25C (Brookhaven National Laboratory). Data processing was performed with *HKL2000* [25]. Molecular replacement was performed with *PHASER* [26] using TEP1*R1 (PDB ID 2PN5) as search model, subsequent refinement in *REFMAC* [27] with model building in *COOT* [28]. Comparison of alternative scenarios in refinement identified the true space group as *P*4_3_ with twinning and rotational pseudosymmetry. The original model of TEP1*R1 (2PN5) was re-refined in *PHENIX* [29] incorporating (i) new TLS groups based on *DYNDOM* [18] analysis TEP1 rigid domain motions and (ii) correction of side chain rotamers by addition of riding hydrogens and analysis by *MOLPROBITY* [30]. TEP1*S1 refinement was completed with TLS, NCS (local) and external restraints derived by *ProSMART* [31] using TEP1*R1 as reference. A representative electron density maps is provided for the TED pre-α4 loop (Figure S1C).

### Accession numbers/ID numbers

TEP1*S1 (PDB 4D93), TEP1*R1 (PDB 4D94).

## Supporting Information

Figure S1 Packing diagram for TEP1*S1 crystals. (A) View along *c* axis showing *P*4_3_ symmetry. (B) View perpendicular to c axis, molecules A and C are shown in yellow, molecule B shown in blue. Molecules B and C are related by pseudotranslational symmetry along *c*, the pseudomerohedral twin law (–*h*,*k*,–*l*) is equivalent to rotation about the 2-fold NCS relating molecules A and C. (C) Representative electron density (1σ) for the pre-α4 loop and Tyr 1275.
(TIF)

Figure S2 Rapid precipitation of TEP1*R1_cut_-C859A. Purified TEP1*R1 and TEP1*R1-C859A full-length protein 1.5 mg/ml (10 µM): (A) before cleavage, and (B) 10 min after addition of 0.5 µM trypsin.
(TIF)

Figure S3 SDS-PAGE analysis of TEP1 precipitation. Silver-stained gels of the soluble fractions of (A) TEP1*S1_cut_ and (B) TEP1*R1_cut_ vs. period of incubation at 20°C.
(TIF)

Table S1 Data Collection and Refinement statistics
(PDF)

Table S2 TEP1*S1 vs. TEP1*R1 superposition of rigid domains
(PDF)

Table S3 Conserved *S*/*R* polymorphisms within the TEP1 thioester domain (TED)
(PDF)

Table S4 Conserved *S*/*R* polymorphisms within the TEP1 MG8 domain
(PDF)

Table S5 Conserved *S*/*R* polymorphisms within the TEP1 CUB domain
(PDF)
