# Supplementary figures and images for: Molecular Basis for Genetic Resistance of Anopheles gambiae to Plasmodium: Structural Analysis of TEP1 Susceptible and Resistant Alleles
Source: PLoS Pathog. 2012 Oct 4;8(10):e1002958. doi: 10.1371/journal.ppat.1002958 (PMC3464232; doi:10.1371/journal.ppat.1002958)

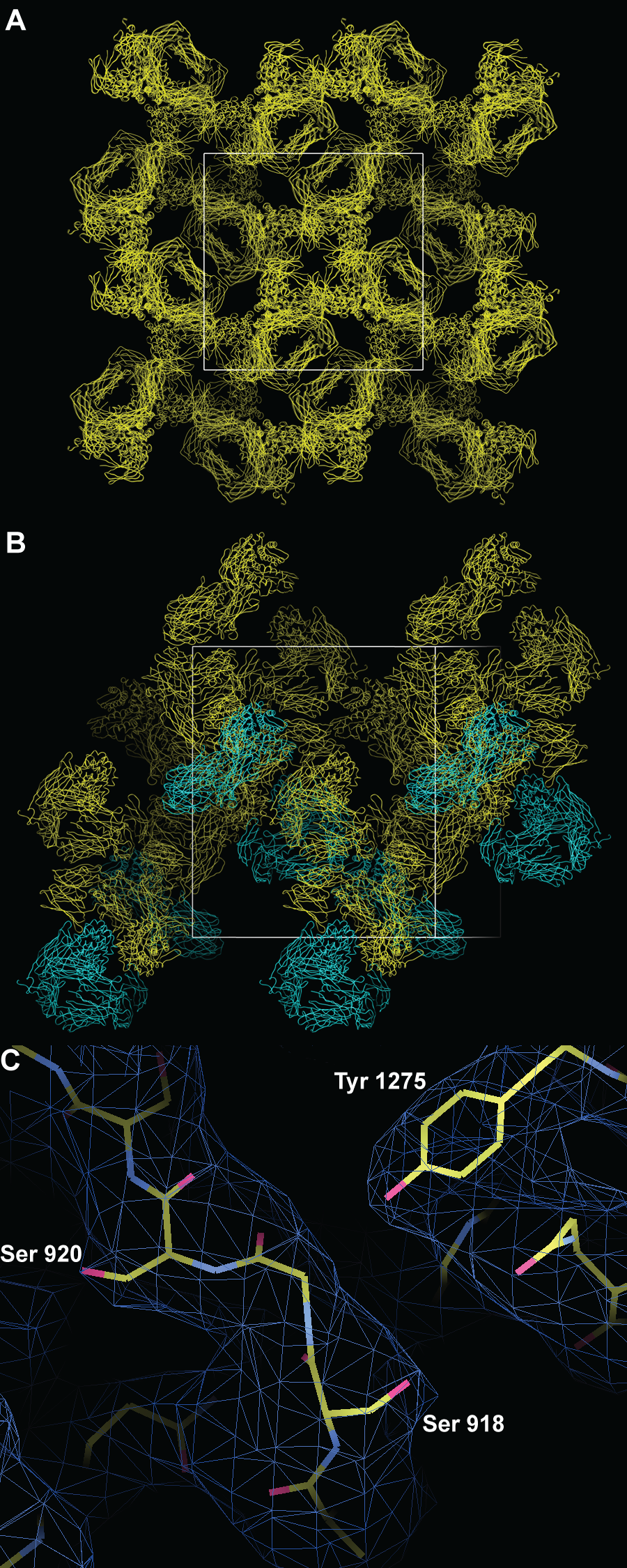

Supplement: Figure S1 — Packing diagram for TEP1*S1 crystals. (A) View along c axis showing P43 symmetry. (B) View perpendicular to c axis, molecules A and C are shown in yellow, molecule B shown in blue. Molecules B and C are related by pseudotranslational symmetry along c, the pseudomerohedral twin law (–h,k,–l) is equivalent to rotation about the 2-fold NCS relating molecules A and C. (C) Representative electron density (1σ) for the pre-α4 loop and Tyr 1275. (TIF) [file ppat.1002958.s001.tif]

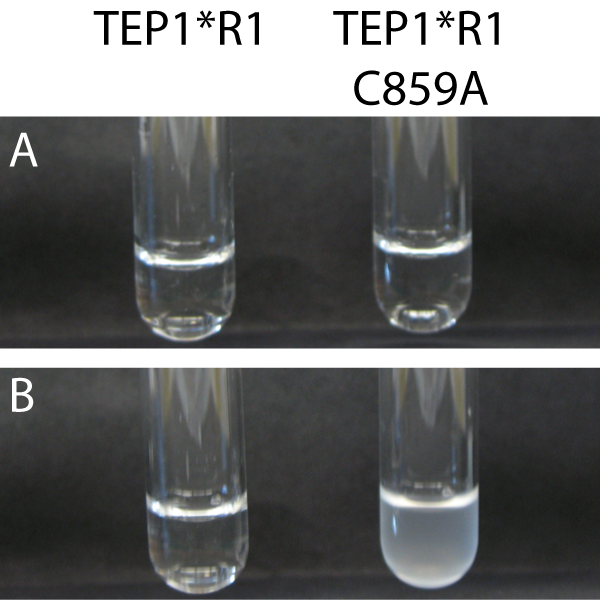

Supplement: Figure S2 — Rapid precipitation of TEP1*R1cut-C859A. Purified TEP1*R1 and TEP1*R1-C859A full-length protein 1.5 mg/ml (10 µM): (A) before cleavage, and (B) 10 min after addition of 0.5 µM trypsin. (TIF) [file ppat.1002958.s002.tif]

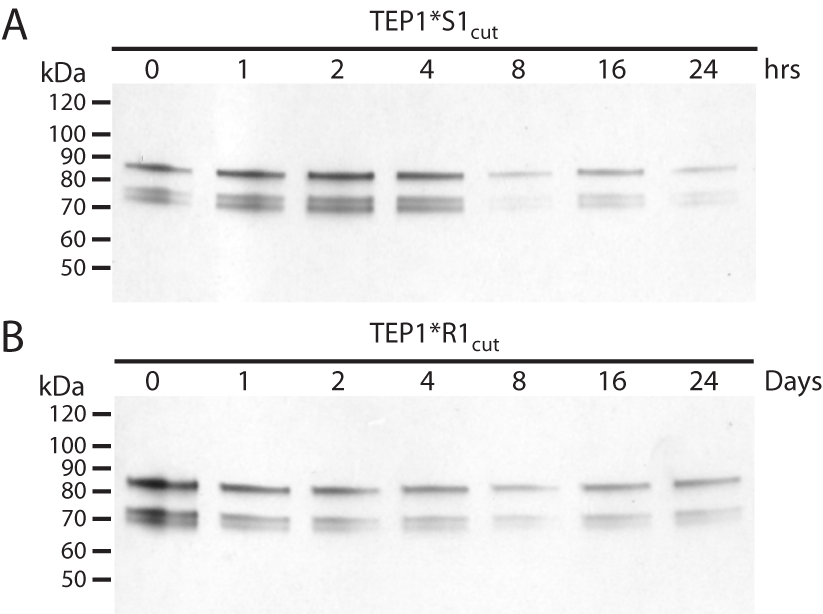

Supplement: Figure S3 — SDS-PAGE analysis of TEP1 precipitation. Silver-stained gels of the soluble fractions of (A) TEP1*S1cut and (B) TEP1*R1cut vs. period of incubation at 20°C. (TIF) [file ppat.1002958.s003.tif]
