# Supplementary material for: Molecular Basis for Genetic Resistance of Anopheles gambiae to Plasmodium: Structural Analysis of TEP1 Susceptible and Resistant Alleles
Source: PLoS Pathog. 2012 Oct 4;8(10):e1002958. doi: 10.1371/journal.ppat.1002958 (PMC3464232; doi:10.1371/journal.ppat.1002958)
Supplement: Table S1 — Data Collection and Refinement statistics (PDF) [file ppat.1002958.s004.pdf]

Table S1: Data Collection and Refinement statistics

	TEP1*S1		TEP1*R1	
Data Collection				
Beamline	NSLS X25C			
Wavelength (Å)	1.1			
Space Group	$P4_3$			
Unit cell (*a*,*b*; *c*) (Å)	196.472; 225.267			
Resolution (Å)	50-3.70 (3.76-3.70)			
Unique reflections	90735 (4503)			
Redundancy	4.9 (5.1)			
Completeness (%)	99.9 (100.0)			
$\langle I \rangle / \langle \sigma \rangle$	22.9 (2.0)			
R_{sym} (%)	7.9 (89.1)			
Refinement				
Resolution (Å)	50.0-3.70 (3.80-3.70)		50.0-2.70 (2.73-2.70)	
Reflections:				
Working set	86136 (6181)		68219 (2250)	
Test set	4583 (331)		3631 (125)	
R_{cryst} (%)	20.0 (30.3)		21.9 (37.7)	
R_{free} (%)	24.0 (35.1)		24.4 (43.1)	
Est. coord. err. (Å)	0.35		0.37	
Rmsd from ideal:				
Bond lengths (Å)	0.015		0.005	
Bond angles (°)	1.785		1.018	
Avg. B-factor ($Å^2$)				
Atoms:				
Non-hydrogen	27047		10343	
Water			133	
Molprobity analysis:				
All-atom clash score	7.29		1.12	
Bad rotamers (%)	6.1	(161/2648)	0.4	(5/1134)
Ramachandran plot:				
Outliers (%)	0.6	(21/3540)	0.3	(5/1264)
Favored (%)	94.4	(3345/3540)	95.9	(1212/1274)
