# Supplementary material for: Molecular Basis for Genetic Resistance of Anopheles gambiae to Plasmodium: Structural Analysis of TEP1 Susceptible and Resistant Alleles
Source: PLoS Pathog. 2012 Oct 4;8(10):e1002958. doi: 10.1371/journal.ppat.1002958 (PMC3464232; doi:10.1371/journal.ppat.1002958)
Supplement: Table S2 — TEP1*S1 vs. TEP1*R1 superposition of rigid domains (PDF) [file ppat.1002958.s005.pdf]

Table S2: TEP1*S1 vs. TEP1*R1 superposition of rigid domains

	Domains	N_{res}	Cα rmsd	residues
1	M3, MG7, CUB, TED, MG8, ANK	744	0.96	222–324, 684–1332
2	MG1, MG2, MG5, MG6	413	1.25	24–217, 423–683
3	MG4	96	2.03	327–422

Domain Pair		Rotation (°)	Translation (Å)	Bending residues
1	2	11.2	0.5	217-222, 683-684
1	3	25.6	6.3	417-423
2	3	22.8	0.3	324-327
