# Supplementary material for: Molecular Basis for Genetic Resistance of Anopheles gambiae to Plasmodium: Structural Analysis of TEP1 Susceptible and Resistant Alleles
Source: PLoS Pathog. 2012 Oct 4;8(10):e1002958. doi: 10.1371/journal.ppat.1002958 (PMC3464232; doi:10.1371/journal.ppat.1002958)
Supplement: Table S3 — Conserved S/R polymorphisms within the TEP1 thioester domain (TED) (PDF) [file ppat.1002958.s006.pdf]

Table S3: Conserved S/R polymorphisms within the TEP1 thioester domain (TED)

Res ID	TEP1*R	TEP1*S	Location	Hum C3	Comment
844<br>851<br>854	T<br>H<br>G	M<br>N<br>A	α0	Q967<br>K979<br>V983	
878<br>880	H<br>I	Y<br>T	α2-3 loop	D1007<br>T1009	
914<br>917<br>918<br>919<br>920	L<br>T<br>T<br>N<br>T	V<br>K<br>S<br>G<br>S	pre-α4 loop	A1046<br>K1049<br>R1050<br>A1051<br>A1072	L914V complements Y950W<br><br><br>*R1*/r^B specific (G in *R2*/r^A)
929	G	A	α4 (buried)	V1061	
936<br>939<br>940	V<br>I<br>S	S<br>M<br>N	α4-5 loop	V1068<br>I1071<br>A1072	*R1*/r^B specific (A in *R2*/r^A)
960	F	S	post-α5 loop	P1092	TEP1R-sTED2 reversion
966	K	E	catalytic loop	E1098	Glu H-bonds to S921 O_γ, F923 N
967	A	T		D1099	
969	A	K		P1101	
970	E	V		V1102	
971	Y	W		I1103	Tyr H-bonds to S921 Oγ
974	E	D	pre-α6 loop	E1106	
991	M	L	α6 (buried)	I1127	
1001	A	V	α6-7 loop (helix)		C3 insertion 1133-42
1005	E	V	α7 face	G1145	TEP1R-sTED2 reversion
1009	K	N		K1149	TEP1R-sTED2 reversion
1012	T	N		D1152	TEP1R-sTED2 reversion
1018	F	L	α7-8 loop	Y1158	
1019	G	A		M1159	
1020	S	F		N1160	
1042	E	K	α9 face	G1182	
1046	N	D		N1186	
1054	F	I	β-hairpin loop	K1193	
1055	I	S			
1057	A	N			
1058	D	N			
1060	N	K			
1063	F	Y		R1197	
1065	N	G		E1199	
1069	P	Q		N1207	
1087	T	L	α10-11 loop	D1225	
1108	S	R	α11-12 loop	S1246	Arg H-bonds to Y1307 O
1122	M	L	α12 (buried)	Y1260	
