# Supplementary material for: Molecular Basis for Genetic Resistance of Anopheles gambiae to Plasmodium: Structural Analysis of TEP1 Susceptible and Resistant Alleles
Source: PLoS Pathog. 2012 Oct 4;8(10):e1002958. doi: 10.1371/journal.ppat.1002958 (PMC3464232; doi:10.1371/journal.ppat.1002958)
Supplement: Table S4 — Conserved S/R polymorphisms within the TEP1 MG8 domain (PDF) [file ppat.1002958.s007.pdf]

Table S4: Conserved S/R polymorphisms within the TEP1 MG8 domain

Res ID	TEP1*R	TEP1*S	Location	Hum C3	Comment
1195	N	H	pre-βA	N1337	
1198	Q	K	βA	D1340	
1215	K	R	βB	E1365	
1219	S	N		R1369	
1223	Q	E		D1373	
1227	R	S	βB-C loop	Q1374	
1228	R	Q		D1375	
1254	K	T	switch region		
1260	K	N	switch region	K1409	H-bonds to Gly 858 O
1261	T	M		Y1410	
1275	D	Y	βC-E loop	D1427	Tyr breaks H-bond to Trp 915
1279	S	T	βC’-E loop	S1432	
1286	L	V	βE (buried)	F1439	
