# Supplementary material for: Molecular Basis for Genetic Resistance of Anopheles gambiae to Plasmodium: Structural Analysis of TEP1 Susceptible and Resistant Alleles
Source: PLoS Pathog. 2012 Oct 4;8(10):e1002958. doi: 10.1371/journal.ppat.1002958 (PMC3464232; doi:10.1371/journal.ppat.1002958)
Supplement: Table S5 — Conserved S/R polymorphisms within the TEP1 CUB domain (PDF) [file ppat.1002958.s008.pdf]

Table S5: Conserved S/R polymorphisms within the TEP1 CUB domain

Res ID	TEP1*R	TEP1*S	Location	Hum C3	Comment
797	V	A	MG7 linker	R915	
800	R	K		K918	
804	R	S	β3-4 strands	R923	
818	T	F		I940	
831	T	K	β4-5 loop	S954	C3 Factor I cleavage site (954)
1142	S	N	β7-8 strands	S1283	*R2*/r[A] and *R1*, C in r[B]
1143	A	T		K1284	
1147	K	N		R1288	
1156	Q	E		L1296	
1159	V	L		S1299	
1160	D	E		E1300	
1183	V	I	MG8 linker	V1323	Factor I cleavage site (1321)
1187	N	D		H1327	
